# Research trends in vascular chips from 2012 to 2022: a bibliometrix and visualized analysis

**DOI:** 10.3389/fbioe.2024.1409467

**Published:** 2024-07-11

**Authors:** Song Yang, Jing Luo, Wanwan Zou, Qikun Zhu, Jianzheng Cen, Qiang Gao

**Affiliations:** ^1^ The Second School of Clinical Medicine, Southern Medical University, Guangzhou, China; ^2^ Department of Cardiovascular Surgery, Guangdong Cardiovascular Institute, Guangdong Provincial People’s Hospital, Guangdong Academy of Medical Sciences, Southern Medical University, Guangzhou, China; ^3^ Guangdong Provincial Key Laboratory of South China Structural Heart Disease, Guangzhou, China; ^4^ School of Medicine South China University of Technology, Guangzhou, China; ^5^ Laboratory of Artificial Intelligence and 3D Technologies for Cardiovascular Diseases, Guangdong Provincial Key Laboratory of South China Structural Heart Disease, Guangdong Provincial People’s Hospital, Guangdong Academy of Medical Sciences, Guangzhou, China

**Keywords:** bibliometric analysis, CiteSpace, VOSviewer, vascular chip, endothelial cells, angiogenesis

## Abstract

**Objective:**

The vascular chip has emerged as a significant research tool, garnering increasing interest and exploration. We utilize bibliometric techniques to analyze literature from the Web of Science (WOS) database, focusing on core journal publications. The aim is to provide a systematic review and prospective outlook on research trends within the vascular chip field, delving into current dynamics and highlighting areas for further investigation.

**Methods:**

We retrieved articles, proceedings papers, and early-access publications related to vascular chips published between January 2012 and December 2022 reported by Web of Science Core Collection (WoSCC) in 2023. Scientific bibliometric analysis was performed using R-bibliometrix, CiteSpace, VOSviewer, and Microsoft Excel software tools.

**Results:**

A total of 456 publications were obtained, including 444 articles, 11 proceedings papers, and one early-access article. These originated from 167 academic journals and 751 research institutions across 44 countries/regions. The United States contributed the majority of publications (41%), with Harvard University leading in contributions (6.6%). Lab on a Chip was the top journal in terms of publications. Notably, authors Jeon NL and Huh D wielded significant influence, with the former being the most prolific author and the latter garnering the most citations. Recent research has predominantly focused on angiogenesis in relation to endothelial cells.

**Conclusion:**

This scientometric investigation comprehensively surveys literature on vascular chips over past decade, providing valuable insights for scholars in the field. Our study reveals global increases in publications, with endothelial cells and angiogenesis being primary research focuses. This trend will persist, drawing continued attention from researchers.

## Introduction

In pursuit of simulating the human tissue environment, Shuler et al. introduced the concept of utilizing chip technology (Esch et al., 2011). Organ on a Chip (OOC) has emerged as a promising platform, enabling the *in vitro* simulation of tissue and organ functions, offering solutions to the limitations of existing cell and animal models in biomedical research (Ronaldson-Bouchard and Vunjak-Novakovic, 2018). Within the realm of OOC, the vascular chip has gained prominence. Researchers have successfully developed various types of vascular chips, including microvascular chips, large vascular chips, and multi-organ chips, facilitating studies involving vascular structure reconstruction, cell-cell and cell-tissue interactions, hemodynamics, endothelial permeability, and thrombosis (Huh et al., 2010; Huh et al., 2011; Kim et al., 2013; Sato and Sato, 2018; Ronaldson-Bouchard et al., 2022; Sarkar et al., 2022). Furthermore, vascular chips have made substantial contributions to drug development, aiding in the prediction of the biological effects of novel drug entities (Cochrane et al., 2019). Angiogenesis and vasculogenesis represent two unique pathways for the formation of blood vessels. Angiogenesis, characterized by the sprouting of new vessels from pre-existing ones, is a process that is typically observed in the phases of embryonic development, wound healing, and tumor progression (Otrock et al., 2007). While vasculogenesis, which involves the *de novo* formation of blood vessels by endothelial progenitor cells, predominantly takes place during the embryonic phase (Risau and Flamme, 1995). And the focus of our study is on angiogenesis.

Bibliometric analysis serves as a powerful method for examining literature characteristics, development trends, and current research focal points. It allows for the evaluation and analysis of various dimensions within the literature, such as quantity, quality, citations, authors, institutions, and keywords, offering insights into a field’s development status and trends (Yang et al., 2021; Yun et al., 2021; Qin et al., 2022; Wan et al., 2022; Guan et al., 2023). In this context, we employed bibliometric analysis, visualization technology, and data mining using authoritative databases to provide a comprehensive overview of developments and research trends in vascular chip research over the past decade. This analysis offers researchers in the field of vascular chips a panoramic view of trends, key institutions, and prominent topics, promoting knowledge sharing, collaboration, and fostering broader advancements to guide future research and innovation.

## Materials and methods

### Data sources and retrieval strategies

In our pursuit of studies pertaining to the vascular chip, we conducted a comprehensive search within the Web of Science Core Collection (WoSCC) at Southern Medical University on 1 November 2023. Our investigation encompassed the period from January 2012 to December 2022. We specifically limited our search to include articles, proceeding papers, and early access publications. Supplementary Figure S1 provides further information about the retrieval formula we employed. Subsequently, we downloaded all retrieved results and subjected their titles and abstracts to a thorough review by two members of our research group, with the aim of meticulously identifying and isolating relevant literature.

### Statistical analysis

From the articles meeting our criteria, we extracted a set of key information, including details such as title, authorship, affiliated research institutions, geographical origin (country or region), keywords, publication year, source, citation counts, and the impact factor (IF) as of 2023. All data records were procured from the WoSCC for subsequent in-depth analysis. To facilitate the processing and visual examination of these variables, we harnessed a combination of tools and software including Microsoft Excel 2020, VOSviewer (version 1.6.20), CiteSpace (version 6.2.R7), and the biblioshiny package within the R programming language. Rstudio was employed to ascertain annual publication output, publication types, prolific countries and regions, notable institutions, prominent journals, and trending keywords. VOSviewer aided us in exploring author-related information and constructing pertinent visual representations of authors, institutions, national cooperation networks, co-cited journals, and co-cited references. CiteSpace allowed us to generate dual-map overlays of journals, conduct clustering analyses of highly-cited literature and co-cited literature, and create timeline distribution maps of clustering. Finally, Microsoft Excel facilitated partial data summarization and table creation for our analytical needs.

## Results

### Annual publications growth trend

**FIGURE 1:**
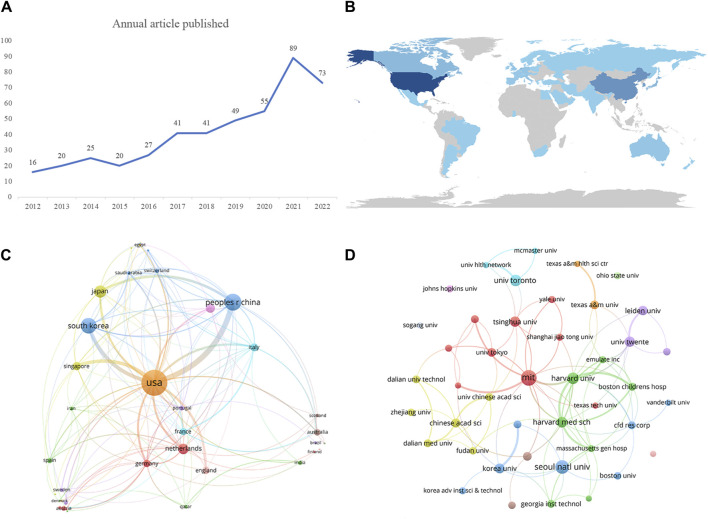
Visualizations illustrating key aspects of vascular chip research. **(A)** Number of articles published per year. **(B)** Network distribution map showcasing the countries involved in publishing vascular chip research, offering insights into the geographical distribution of contributions. **(C)** Network map displaying collaborations between different countries in the field, highlighting international cooperation and partnerships. **(D)** Network map specifically focused on institutions engaged in Vascular chip research, with node sizes reflecting their publication volumes.

In our analysis, we retrieved a total of 456 articles, which encompassed 11 proceedings papers, and one early access article, with an average annual publication rate of 41 articles. Figure 1A illustrates the yearly output of literature related to vascular chip research, showing an upward trend from 2012 to 2022. Although there were declines in article numbers in 2015 and 2022, the overall activity in the field of vascular chip research remained relatively high. The lowest publication count was in 2012, while the highest was recorded in 2021. Notably, in 2020, there was a peak annual growth rate of 61.81%, suggesting rapid development in vascular chip research during that period.

### Country/region and institution analysis

Our analysis involved 751 institutions from 44 countries/regions that collaborated in co-publishing the 456 publications. Figure 1B provides a visual representation of the distribution of publications worldwide, with darker colors indicating higher publication numbers. Among all the regions, European countries contributed the most publications. Table 1 ranks the top 10 countries/regions and institutions by publication volume. The United States led among the countries, with a total of 187 papers, closely followed by China with 91 publications. Additional details on the top ten countries/regions and their respective publication counts are available. Additionally, we present a network map illustrating country collaborations in Figure 1C.

**TABLE 1:** The top 10 countries/regions in terms of the number of publications and the number of citations.

Rank	Country/region	Publications	Citations	Average article citations
1	The United States	187	11,093	59.32
2	China	91	1,811	19.90
3	South Korea	69	2,917	42.27
4	Japan	42	1,509	35.93
5	Netherland	33	1,365	41.36
6	Canada	26	1,118	43.00
7	Singapore	21	831	39.57
8	Italy	18	564	31.33
9	Germany	17	896	52.71
10	England	17	714	42.00

Table 2 provides an overview of the top 10 publishing institutions in terms of publication quantity. These institutions are spread across five countries/regions, namely the United States, China, Netherlands, South Korea, and Canada. Additionally, we specifically selected institutions with more than 10 publications to construct a collaborative network, as depicted in Figure 1D. In this visual representation, the nodes representing the Massachusetts Institute of Technology and Seoul National University are notably larger than others due to their higher research publication volumes. Furthermore, it is evident that numerous positive collaborations exist among different institutions. For instance, the Massachusetts Institute of Technology maintains close partnerships with Harvard University, Harvard Medical School (affiliated with Harvard University), Tsinghua University, and the University of Tokyo.

**TABLE 2:** The top 10 publishing institutions in the number of publications.

Rank	Institution	Count	Citations
1	Massachusetts Institute of Technology	29	2,442
2	Seoul National University	23	1,596
3	Harvard University	16	1,706
4	University of Toronto	16	446
5	Harvard Medical School	14	1,155
6	University of Twente	13	836
7	Tsinghua University	13	232
8	Korea University	12	289
9	Leiden University	11	436
10	Chinese Academy of Sciences	10	92

### Journals and highly cited academic journals

Between January 2012 and December 2022, a total of 167 academic journals contributed to the publication of 456 papers in the field of vascular chip research. Table 3 highlights the top 10 journals, with *Lab on a Chip*, a prestigious journal in micro-nano engineering published by the Royal Society of Chemistry, leading with 66 articles (Impact Factor 2022 = 6.1, Q1). A visual analysis of citation numbers in these journals is presented in Table 3. *Lab on a Chip* received citations exceeding 1,000 times in several journals, with a total of 1,502 citations. This was followed by the *P Natl Acad Sci* USA journal, with 692 citations. These findings indicate that *Lab on a Chip* holds a prominent position both in terms of publication and citation counts in the vascular chip research field. Additionally, a visual representation of vascular chip-related journal overlay is provided in Supplementary Figure S2.

**TABLE 3:** The top 10 journals in the number of publications and their citations, IF, and JCR partitions.

Rank	Journals	Count	IF2022	Q	Co-cited journals	Citation	IF2022	Q
1	Lab on A Chip (UK)	66	6.1	Q1	Lab on A Chip (UK)	1,502	6.1	Q1
2	Biomicrofluidics (USA)	21	3.2	Q2	P Natl Acad Sci (USA)	692	11.1	Q1
3	Scientific Reports (UK)	18	4.6	Q2	Biomaterials (Netherlands)	595	14	Q1
4	Micromachine (Switzerland)	15	3.4	Q2	PLos One (USA)	451	3.7	Q2
5	Advanced Healthcare Materials (Germany)	13	10	Q1	Scientific Reports (UK)	447	4.6	Q2
6	Biomedical Microdevices (Netherlands)	12	2.8	Q3	Nature (UK)	360	64.8	Q1
7	Biomaterials (Netherlands)	11	14	Q1	Analytical chemistry (USA)	277	7.4	Q1
8	Analytical chemistry (USA)	10	7.4	Q1	Science (USA)	266	56.9	Q1
9	Biotechnology and bioengineering (USA)	9	3.8	Q2	Blood (USA)	262	20.3	Q1
10	Frontiers in Bioengineering and Biotechnology (USA)	9	4.8	Q2	Cell (United States)	825	64.5	Q1

**FIGURE 2:**
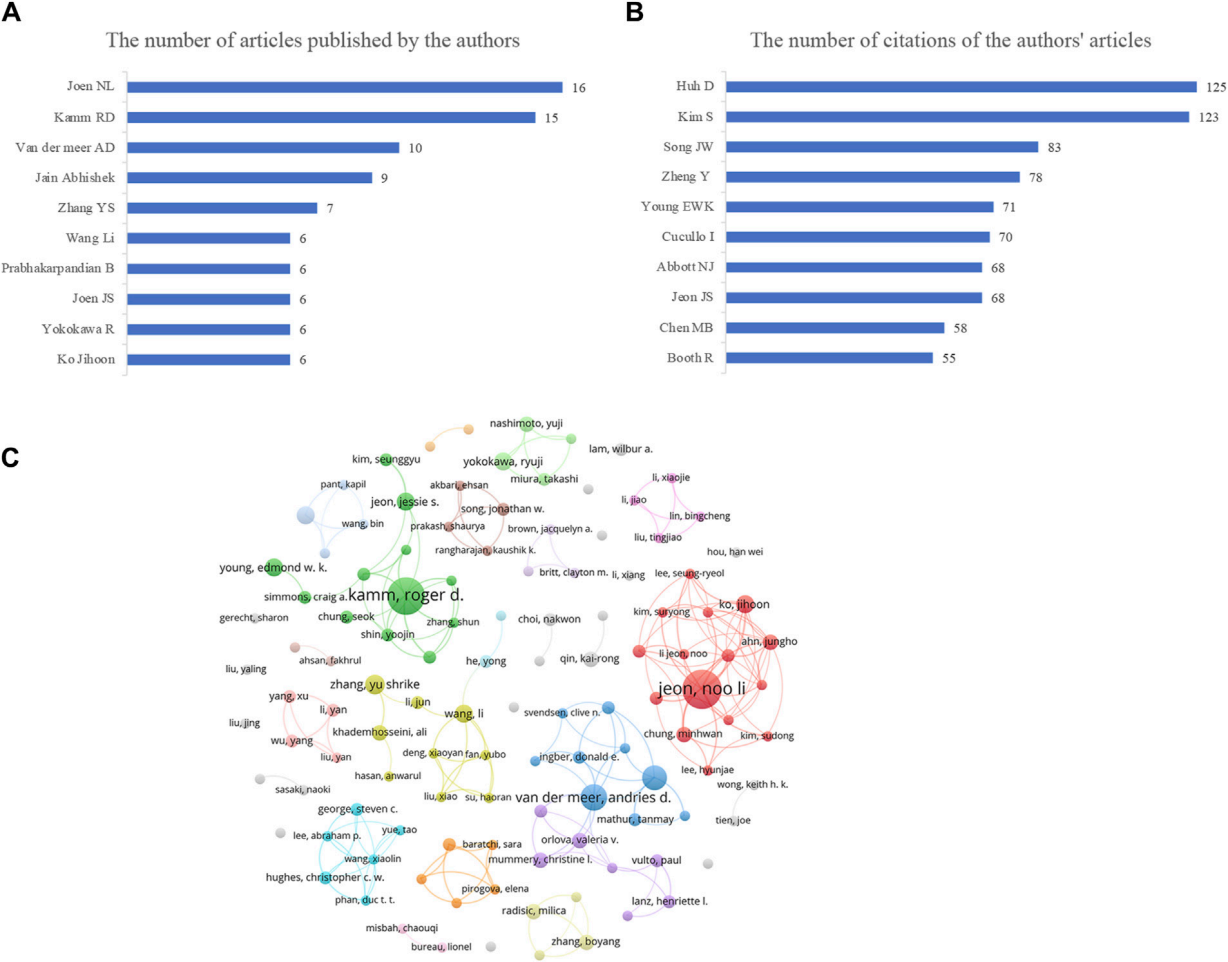
Visualizations of the authors and their contributions in vascular chip research. **(A)** Number of articles published by individual authors, offering insights into the productivity of different researchers in the field. **(B)** Total number of citations of articles published by the authors reflects the degree of influence of different authors in the research field. **(C)** Author cooperation network diagram, illustrating collaborations among authors in the vascular chip research domain, showcasing the connections and partnerships among researchers.

Our analysis identified 2,526 authors involved in Vascular chip research. Figure 2A presents the top 10 authors in this field between January 2012 and December 2022. Jeon NL emerged as the most prolific author with 16 published articles, followed by Kamm RD with 15 articles and Van der Meer AD with 10 articles. To assess the impact of their work, we selected the top 10 authors with the highest citation counts (Figure 2B). Huh D received the highest total citations at 125, followed by Kim S with 123 citations. Figure 2C displays an author cooperation network diagram for authors who published more than three papers, where larger nodes indicate more publications and connections represent collaborations. We observed that both researchers, Kamm RD and Jeon NL, have distinguished themselves through their respective contributions to collaborative research. To further illustrate their collaborative work, we have included their individual collaboration network diagrams. Although Huh D and Kim S published fewer articles, their high citation counts may suggest significant research quality, and the methods they used might be worth our reference and emulation. Despite the fact that our current analysis has been conducted using high citation frequency as the sole criterion, the findings of our analysis are aimed at providing a reference for researchers intrigued by this field of study, with the goal of fostering further academic discourse and comprehension within the domain. By sharing our insights, we aspire to ignite the curiosity of fellow researchers, motivating them to delve into and utilize these exemplary research approaches, thereby propelling the advancement of the field.

### Co-cited references

Co-citation denotes the occurrence where two or more distinct articles or documents are concurrently referenced within the same reference list of another publication. This is a common occurrence when scholars authoring works on analogous subjects draw upon a diverse array of sources to substantiate their research. Co-citation serves as a method to illustrate the interconnections between various scholarly endeavors. Supplementary Table S1 lists the top 10 co-cited articles, most of which are related to angiogenesis and have been cited more than 30 times, with four articles exceeding 50 citations. Notably, Kim S’s study titled “Engineering of functional, perfusable 3D microvascular networks on a chip” received the highest number of citations at 77. Literature with 20 or more co-citations was used to construct a co-citation map (Supplementary Figure S3A). Cluster analysis revealed the seven largest clusters, with “microfluidic platform,” “microfluidic chip,” and “ischaemic stroke” being the top three with the widest scope (Supplementary Figure S3B). A visual analysis of these clusters over time is presented in Supplementary Figure S4, showing that “cell transmigration” and “tissue engineering” were early areas of vascular chip research, with current hotspots including ‘microfluidic,” “blood-brain barrier,” “bioengineering,” and “bioprinting.”

### References with citation burstiness

Citation bursts indicate periods of intense attention to specific literature in a field. CiteSpace identified eight articles with strong citation bursts in vascular chip research, where the shortest publication time was set to 4 years. Supplementary Figure S5 illustrates the extent of these bursts, with 2013 being a particularly significant year, accounting for 38.1% (8/21) of sudden citations. Among these, Zheng Y’s work titled “*In vitro* microvessel for the study of angiogenesis and thrombosis” led with a burst strength of 11.21, primarily during 2013–2017. The second article with a strong burst (Strength = 10.04) was “Engineering of functional, perfusable 3D microvascular networks on a chip” by Kim S’s team in *Lab on a Chip*, which experienced a burst of citations from 2014 to 2018. These two articles also exhibited strong citation bursts between 2018–2022, indicating their recent high research quality and interest among scholars.

### Analysis of hot keywords

**FIGURE 3:**
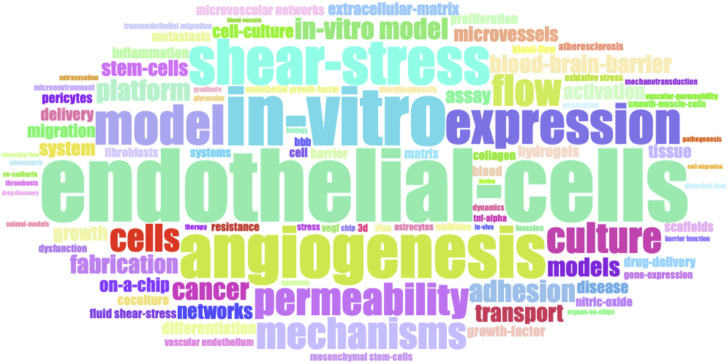
Hot keywords visualized in a word cloud.

A word cloud map (Figure 3) based on author keywords in the literature reveals the frequency of keywords. Larger nodes indicate higher keyword frequency. Notably, the top five keywords are “endothelial cells,” “*in-vitro*,” “angiogenesis,” “shear-stress,” and “expression.” This suggests that over the past decade, researchers have focused on using vascular chips for studies involving endothelial cells. Additionally, other research directions, such as constructing *in vitro* vascular network models, exploring angiogenesis, assessing the effects of fluid shear stress on vascular cell growth, and studying permeability, all relate to endothelial cells.

## Discussion

### General information

Here, we undertook a bibliometric analysis of publications pertaining to vascular chips over the past decade. Our objective was to identify key trends and focal points in the realm of vascular chip research. Our findings revealed a consistent growth in the number of annual publications within this field from 2012 to 2022. Notably, the year 2020 saw the highest annual growth rate, reaching an impressive 61.81%. This data suggests that vascular chip research will continue to captivate the interest of scholars and researchers in the forthcoming years.

In terms of productivity, the United States emerged as the most prolific country with 187 articles published during this period, followed by China with 91 articles and South Korea with 69 articles. American researchers contributed to approximately 41.0% of all publications, solidifying the United States as being in a dominant position in terms of publication volume, citations, and international collaborations. This prominence indicates a significant role for the United States in advancing vascular chip research. It is worth noting that despite Germany’s lower publication count, their research demonstrated higher citation rates, highlighting the quality of their contributions. This phenomenon highlights the importance of international cooperation in promoting knowledge exchange and improving research quality, especially with top contributors like the United States.

Examining the core journals in this field, *Lab on a Chip* emerged as a frontrunner. Not only did *Lab on a Chip* lead in the number of published papers, it also boasted 1,502 citations. Over the ten-year span from 2012 to 2022, the journal published a total of 66 articles, making significant academic contributions. It is noteworthy that the scope of *Lab on a Chip* extends beyond vascular chips, encompassing various organ chips. This versatility speaks to its appeal across diverse research fields and offers a broad academic platform for vascular chip researchers.

Analyzing authorship, Jeon NL and Kamm RD emerged as the most prolific authors with the highest number of published papers and citations. Jeon NL hails from Seoul National University, while Kamm RD is affiliated with MIT. Both authors have primarily focused their vascular chip research on constructing *in vitro* microvascular models to explore angiogenesis mechanisms and diseases related to blood vessels. Recent work by Kamm RD (Wan et al., 2023) demonstrates the design of a vascular chip that combines modern biomaterials and 3D cell culture to simulate angiogenesis in tumor spheres. Co-cultivating tumor spheres with fibroblasts within the microfluidic chip led to an increase in angiogenesis. Moreover, under continuous flow conditions, angiogenesis was further enhanced. Additionally, the researchers introduced immune cell-chimeric antigen receptor T-cells (CAR-T cells) known for their antitumor activity. Interestingly, the presence of CAR-T cells was observed to stimulate angiogenesis within the tumor spheres. Jeon NL’s research (Lim et al., 2023), on the other hand, centers on a human cerebrovascular choroid plexus chip to study cancer cell penetration of the cerebrovascular wall. These innovative approaches provide valuable insights into angiogenesis processes and interactions between cells in different physiological contexts.

Lastly, our analysis of references and keywords underscores the central role of vascular endothelial cells in vascular chip research. A significant portion of the literature focused on utilizing vascular endothelial cells to construct *in vitro* vascular network models, investigate angiogenesis, and explore the impact of fluid shear stress on vascular cell growth. For instance, Kim S’s team employed a microfluidic chip to co-culture human umbilical vein endothelial cells (HUVECs) and lung fibroblasts, simulating angiogenesis network formation and cell interactions (Kim et al., 2013). Their research highlighted the influence of pro-angiogenic factors secreted by lung fibroblasts on angiogenesis in HUVECs and the enhancement of endothelial barrier stability under simulated blood flow shear stress. In their research, they discovered that pro-angiogenic factors released by lung fibroblasts have the capability to trigger the development and expansion of angiogenesis buds within HUVECs It has also been reported that cancer-associated fibroblasts and human skin fibroblasts can promote angiogenesis (Pauty et al., 2021; Bae et al., 2023). Furthermore, when simulating *in vivo* blood flow shear stress stimulation (at approximately 0.31–7.22 dyne/cm^2^), vascular endothelial cells exhibited a close alignment along the direction of fluid flow, effectively reinforcing the stability of the endothelial barrier. Similarly, Zheng Y’s team utilized vascular chips to inoculate HUVECs into a type I collagen matrix, resulting in the construction of an *in vitro* microvascular network (Zheng et al., 2012). Their work delved into angiogenesis processes, endothelial-perivascular cell interactions, and blood-endothelial cell interactions, and illustrated that the presence of perivascular cells led to tighter and more stable endothelial cell connections.

Taken together, our analysis of vascular chip research demonstrates its significance, particularly in understanding angiogenesis mechanisms and the role of vascular endothelial cells. This field has exhibited steady growth, with prominent contributions from the United States, Korea, and China, as well as leading institutions and prolific authors. The journal *Lab on a Chip* has played a pivotal role, showcasing its versatility across multiple organ chip disciplines. Enhanced international cooperation and the exploration of diverse research avenues are crucial to advancing the field of vascular chips.

### Emerging topics

In the realm of emerging topics within Vascular chip-related research, we have compiled a list of 21 references exhibiting strong citation bursts, reflecting sustained interest among researchers. After combing through these articles, we found that the theme evolution of vascular chips can be roughly divided into three research stages. First, the rise of vascular chip technology provides a powerful *in vitro* research platform for researchers to explore the interaction between and migration of vascular cells. To better simulate the physiological environment of blood vessels *in vivo*, researchers have begun to work on adding fluid perfusion on the chip to approximate the fluid shear stress on blood vessels. On this basis, researchers can explore angiogenesis and its influencing factors in order to more completely construct a perfusable vascular network on the chip. With the success of the construction of a perfusion vascular network, researchers have further investigated the construction of a perfusion blood-brain barrier (BBB) chip to understand its barrier function and perform drug screening. Researchers have also used vascular chips to study the mechanism of tumor cell penetration in blood vessels. In general, researchers are constantly improving the vascular chip to more accurately simulate the physiological environment of blood vessels *in vitro* and are constantly expanding the research of other vascular-related diseases.

These references can be divided into two groups: the first eight references, with citation intensity from 2012 to 2016, and the subsequent two references, with citation intensity spanning from 2018 to 2022. Notably, the three most cited articles within this collection were authored by Zheng Y (Strength = 11.21), Kim S (Strength = 10.04), and Bhatia SN (Strength = 9.04). In their review article Bhatia SN (Bhatia and Ingber, 2014) highlighted that microfluidic chips offer distinct advantages over traditional 2D or 3D culture systems when it comes to simulating physiological aspects of tissues and organs. They excelled in reproducing multicellular structures, tissue-tissue interfaces, physical and chemical microenvironments, and vascular perfusion akin to the human body. This technology holds value for studying molecular mechanisms of action, lead candidate prioritization, toxicity testing, and biomarker identification. Although these chips may not fully replicate the intricate human body environment due to technological limitations, they nonetheless contribute significantly to the drug discovery and development process by providing more accurate and efficient screening methods and aiding in understanding the roles of human tissues and organs in basic biology and pathobiology.

Recent years have seen the emergence of strong citation bursts by authors Wang YI (Strength = 3.81) and Kim Seunggyu (Strength = 2.61), with continuous citation strength from 2018 to 2022. Wang YI’s team successfully developed a high-fidelity microfluidic BBB model using human induced pluripotent stem cell (hiPSC)-derived brain microvascular endothelial cells (BMECs) co-cultured with rat primary astrocytes (Wang et al., 2017). This model effectively simulates the characteristics of the *in vivo* BBB over an extended period, achieving the highest transendothelial resistance of up to 4,000 Ω cm^2^. Importantly, the model demonstrated drug permeability similar to that observed *in vivo*, presenting a valuable tool for candidate drug screening.

Kim Seunggyu’s article (Kim et al., 2017) titled “Vasculature-on-a-chip for *in vitro* disease models,” offers an insightful review of various methods for vascularization on microfluidic chip platforms. These methods include cell patterning, sacrificial molds, patterned microchannels, and self-assembly. The article comprehensively analyzes the mechanical, chemical, and biological factors contributing to vascularization on these chips. It delves into applications spanning endothelial dysfunction, tumor angiogenesis, cancer metastasis, BBB, the lymphatic system, and drug screening for vascular diseases.

### Strengths and limitations

This research presents several notable strengths. First, it represents the inaugural use of scientometric methods to systematically analyze vascular chip-related literature, providing valuable guidance for clinicians and scholars interested in this field. Secondly, we employed three scientometric tools, ensuring objectivity in our data analysis. Moreover, compared to traditional narrative reviews, scientometric analysis offers deeper insights into evolving research focuses and trends.

However, our study does have certain limitations. First, we did not make a clear distinction between “vasculogenesis” and “angiogenesis” in our search criteria. Although both “vasculogenesis” and “angiogenesis” are utilized in some vascular chip experiments for the study of new blood vessel formation, they have different mechanisms. However, in our actual work, we found that even after supplementing the search criteria with “vasculogenesis,” the final search results showed no difference. For bibliometric analysis articles, this implies that our final analysis results are unaffected, and the results may even include some studies on “vasculogenesis”. Second, due to the inclusion of cited paper that may encompass organ-on-chip technology related literature rather than those directly related to vascular-on-chip, the results might have been overestimated. Third, we exclusively retrieved data from WoSCC without utilizing additional databases. Web of Science, managed by Clarivate Analytics, is renowned for its rigorous selection criteria for journals. While its journal coverage is more focused, it is widely regarded as a repository of high-quality scholarly work. Scopus, managed by Elsevier, and Google Scholar, a versatile web-based search engine for academic literature, both offer more extensive journal coverage than Web of Science. However, the capabilities of bibliometric analysis tools for processing data from Scopus and Google Scholar are somewhat restricted. Moreover, the indexing accuracy and completeness of Google Scholar may vary due to the dynamic nature of web links, and the absence of a defined selection process can result in a variable quality of indexed documents. Taking into account the pros and cons of each database, we have chosen Web of Science for our comprehensive analysis to ensure a thorough and methodologically sound evaluation of our search results. Additionally, the limitations inherent in existing scientific measurement tools may have introduced some bias into our results. For instance, the possibility of author name duplications cannot be entirely ruled out, as these tools do not provide precise data on this matter.

## Conclusion

Utilizing bibliometrics and databases like CiteSpace, VOSviewer, R-bibliometrics, and Microsoft Excel, we conducted a comprehensive analysis of the knowledge base and research hotspots in the vascular chip field over the past decade. Our findings spotlighted specific countries, institutions, authors, and journals that have significantly contributed to this research domain. Our study underscores the promising prospects within vascular chips, with related literature demonstrating a consistent growth trajectory. Currently, angiogenesis stands out as a key research focus in this field, with tumor metastasis and BBB studies emerging as potential areas of interest deserving further exploration. As researchers delve deeper into vascular chips, we anticipate this technology will continue to evolve, becoming increasingly accurate and efficient in simulating microenvironments, exploring physiological mechanisms, conducting drug testing, and screening candidates.

## Supplementary material

SUPPLEMENTARY FIGURE S1 The screening process of the literature retrieved from the WoSCC.

SUPPLEMENTARY FIGURE S2 Dual-map overlay of articles citing vascular chip research. The data display two primary reference paths. The figure illustrates that the published papers predominantly cluster within journals related to molecular biology, immunology, and physics, as well as materials and chemistry. On the other hand, the cited papers are primarily concentrated within journals in the fields of molecular biology, genomics, chemistry, materials, and physics. This visual representation highlights the interdisciplinary nature of vascular chip research, drawing from various scientific domains.

SUPPLEMENTARY FIGURE S3 Visualizations of co-cited references and clustering analysis of cited literature. **(A)** Network map of co-cited references, illustrating the interconnections among references that are frequently cited together in vascular chip research, revealing key knowledge clusters. **(B)** Analysis of the largest seven clusters within the cited literature, identifying thematic groupings and areas of concentrated research interest within the field.

SUPPLEMENTARY FIGURE S4 Visual clustering time axis analysis diagram of vascular chip research. This diagram illustrates how research themes, topics, or clusters within the field have evolved and changed over time. These data may help identify trends, shifts in focus, and the chronological development of key research areas within the domain of vascular chip research.

SUPPLEMENTARY FIGURE S5 Top 21 references with the strongest citation bursts.
