# Supplementary figures and images for: Research trends in vascular chips from 2012 to 2022: a bibliometrix and visualized analysis
Source: Front Bioeng Biotechnol. 2024 Jul 11;12:1409467. doi: 10.3389/fbioe.2024.1409467 (PMC11269249; doi:10.3389/fbioe.2024.1409467)

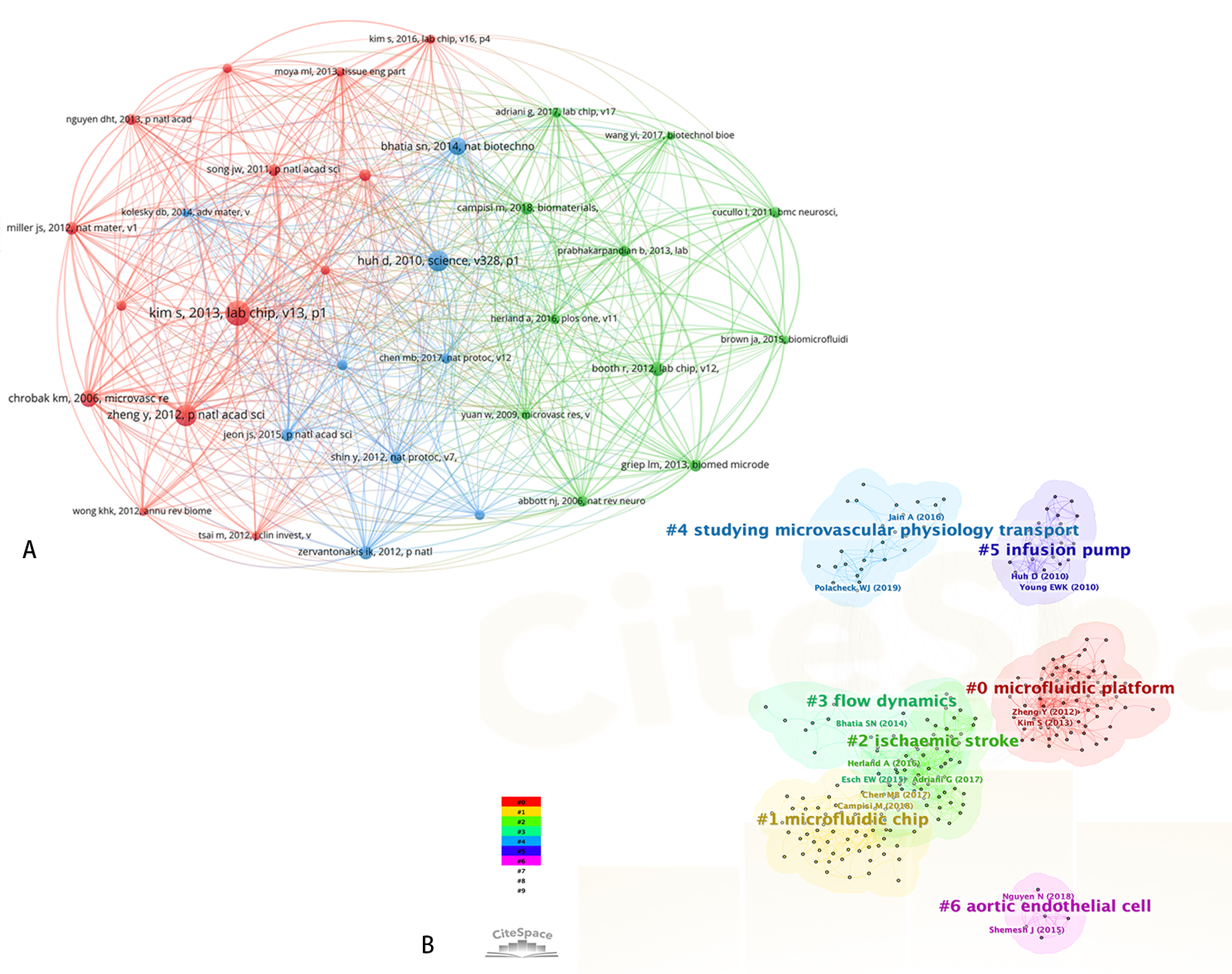

Supplement: Supplementary file 1 [file Image3.TIF]

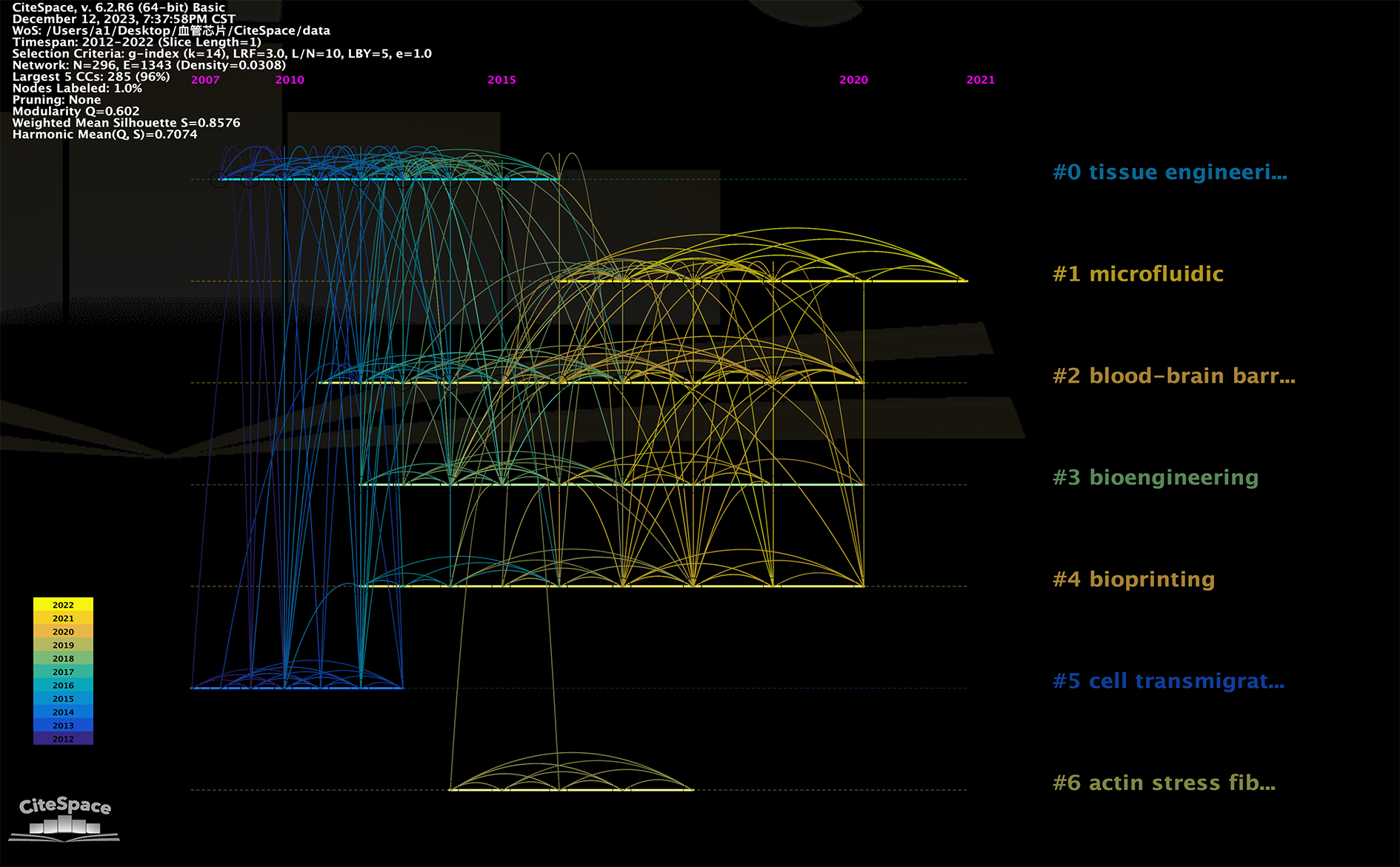

Supplement: Supplementary file 2 [file Image4.TIF]

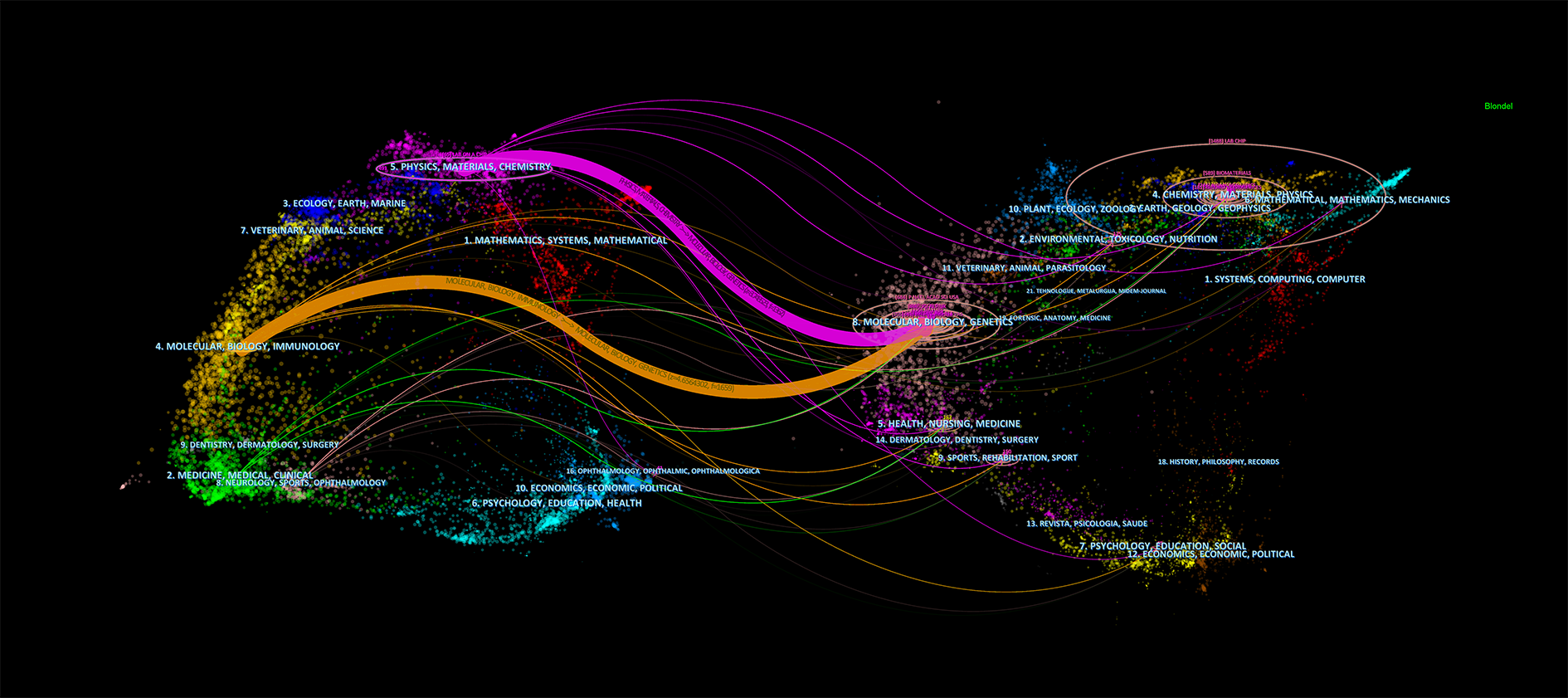

Supplement: Supplementary file 3 [file Image2.TIF]

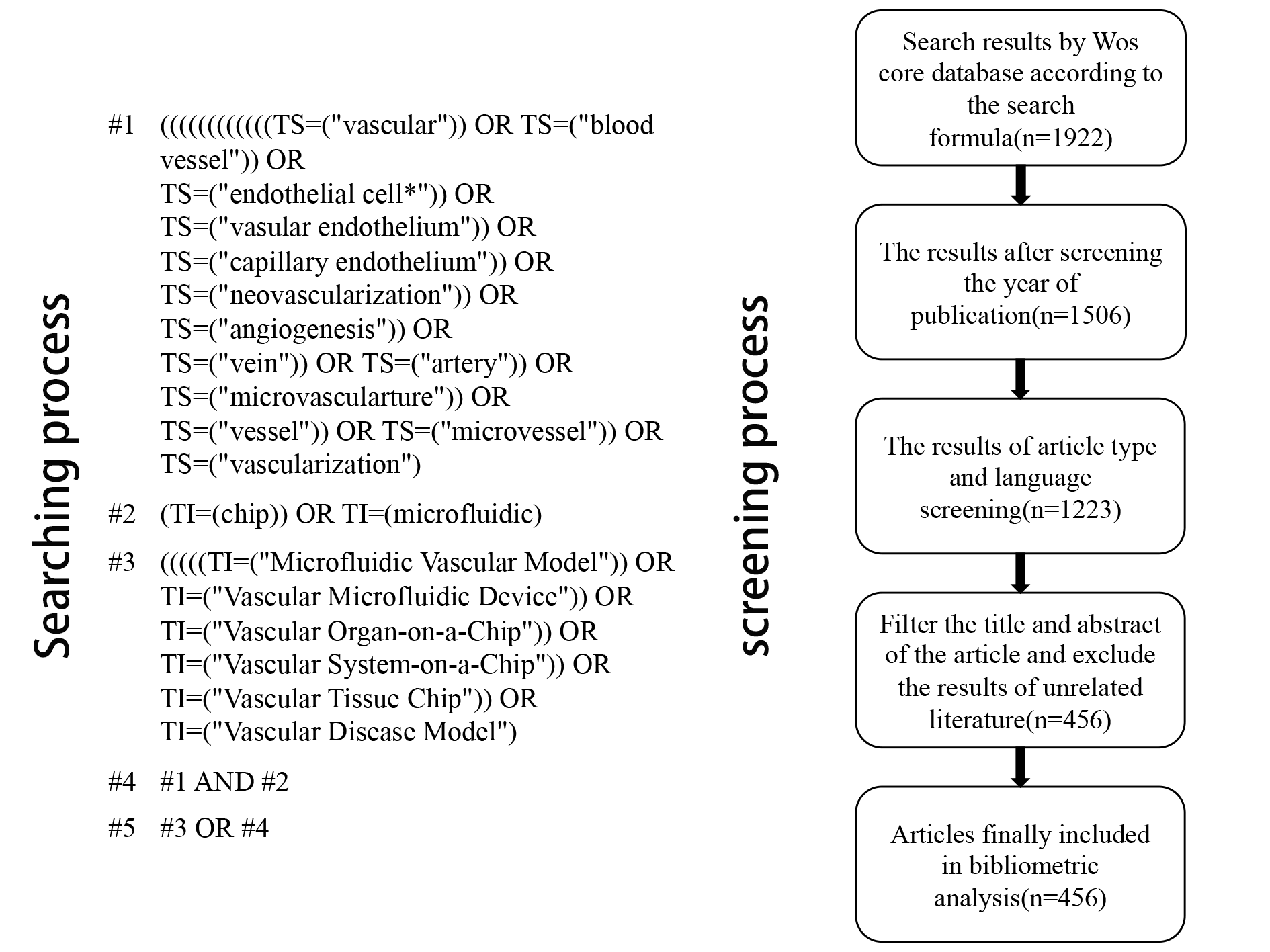

Supplement: Supplementary file 4 [file Image1.TIF]

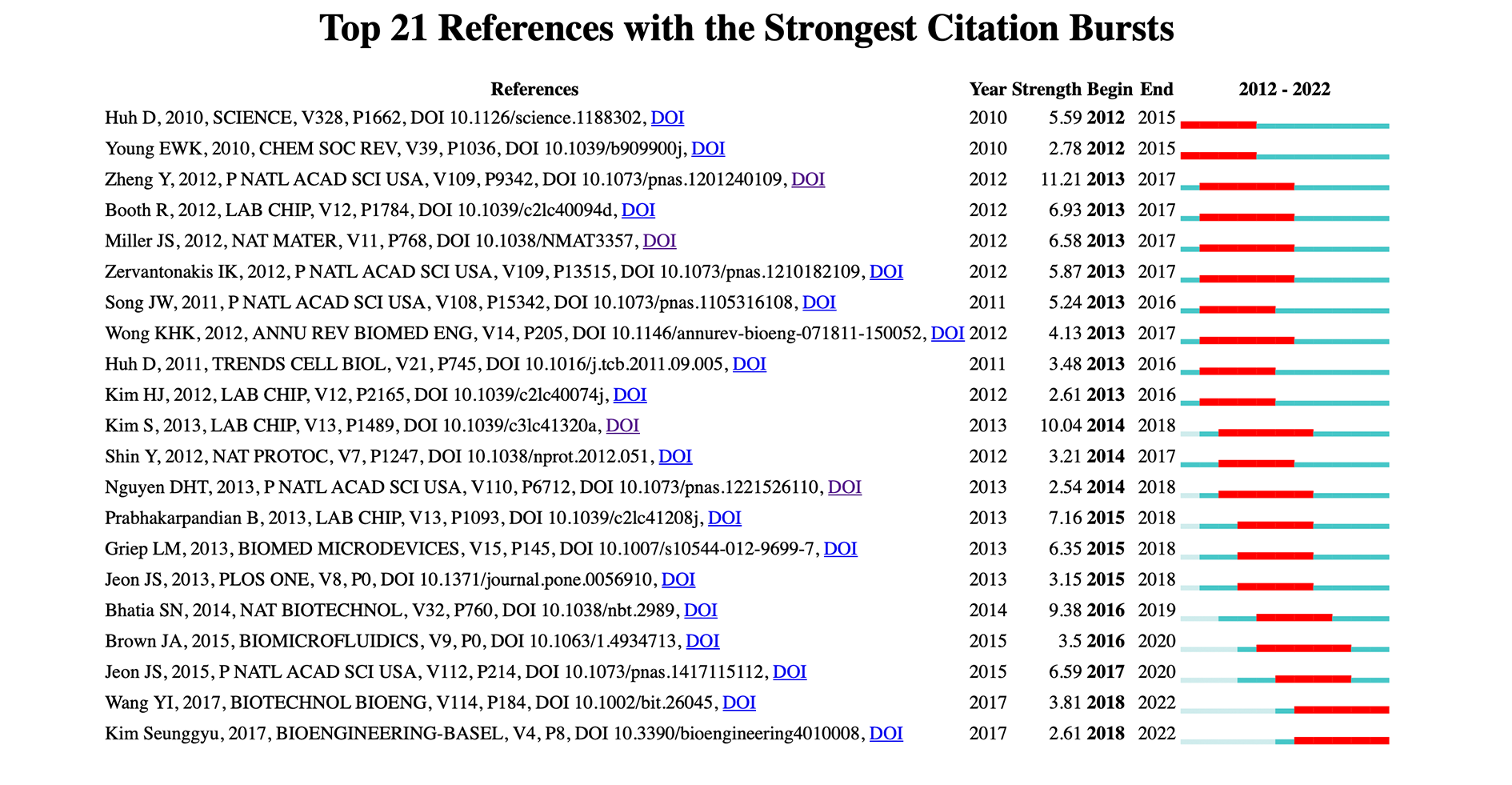

Supplement: Supplementary file 6 [file Image5.TIF]
