# Supplementary material for: Research trends in vascular chips from 2012 to 2022: a bibliometrix and visualized analysis
Source: Front Bioeng Biotechnol. 2024 Jul 11;12:1409467. doi: 10.3389/fbioe.2024.1409467 (PMC11269249; doi:10.3389/fbioe.2024.1409467)
Supplement: Supplementary file 5 [file Table1.DOC]

**Table S1.** The top 10 co-cited articles of vascular chip research.

| ***Rank*** | ***Co-cited reference*** | ***Count*** | ***Cell type*** |
| --- | --- | --- | --- |
| *1* | *Kim S, 2013, Lab Chip, v13, p1489, DOI: 10.1039/c3lc41320a* | *77* | *HUVECs* |
| *2* | *Zheng Y, 2012, P Natl Acad Sci USA, v109, p9342, Doi 10.1073 /Pnas. 1201240109* | *63* | *HUVECs* |
| *3* | *Huh D, 2010, science, v328, p1662, Doi 10.1126/science.1188302* | *60* | *HPMECs* |
| *4* | *Bhatia SN, 2014, Nat Biotechnol, v32, p760, Doi 10.1038/Nbt.2989* | *51* | *\* |
| *5* | *Chrobak KM, 2006, microvasc res, v71, p185, Doi 10.1016/j.mvr.2006.02.005* | *48* | *HUVECs、HDMECs* |
| *6* | *Booth R, 2012, Lab Chip, v12, p1784, doi 10.1039/c2lc40094d* | *39* | *b.End3* |
| *7* | *Zervantonakis Ik, 2012, P Natl Acad Sci USA, v109, p13515,Doi10.1073/Pnas.1210182109* | *36* | *Primary MVEC、HUVECs* |
| *8* | *Campisi M, 2018, Biomaterials, v180, p117, Doi 10.1016 /j.Biomaterials.2018.07.014* | *34* | *hiPSC-ECs* |
| *9* | *Jeon JS, 2015, p Natl Acad Sci USA, v112, p214, Doi 10.1073/Pas. 1417115112* | *34* | *HUVECs* |
| *10* | *Miller JS, 2012, Nat Mater, v11, p768, Doi [10.1038/nmat3357 10.1038/nmat3357]* | *32* | *HUVECs* |

*The data within Table S1 is derived from the references cited by authors in their respective published studies, which form the foundation of our analytical findings. This implies that the article subject to co-citation could either be a review article or an original research paper. Consequently, the data showcased in Table S1 encompasses more than just the articles that have been specifically searched for and selected by us.
